# The political economy of novel food taxes and subsidies in India: insights from stakeholder interviews and a scoping review

**DOI:** 10.1093/heapol/czag093

**Published:** 2026-07-21

**Authors:** Amit Summan, Divija Samria, Seshadri Dutta, Ramanan Laxminarayan

**Affiliations:** One Health Trust, 5636 Connecticut Avenue NW, PO Box 42735, Washington, DC 20015, United States; Economics for Health, Johns Hopkins Bloomberg School of Public Health, Baltimore, MD 21205, United States; One Health Trust, Obeya Pulse, First Floor, 7/1, Halasur Road, Bengaluru, Karnataka 560042, India; One Health Trust, Obeya Pulse, First Floor, 7/1, Halasur Road, Bengaluru, Karnataka 560042, India; One Health Trust, Obeya Pulse, First Floor, 7/1, Halasur Road, Bengaluru, Karnataka 560042, India; High Meadows Environmental Institute, Princeton University, Princeton, NJ 08544, United States

**Keywords:** health taxes, nutrition-based food subsidies, nutrition-based fiscal policies, non-communicable diseases, political economy analysis, India

## Abstract

Fiscal policy tools are cost-effective for curbing unhealthy consumption, but unlike the extensively studied cases of tobacco and alcohol, the political economy of nutrition-based taxes and subsidies remains poorly understood in low- and middle-income countries. We examined the political economy of factors influencing the adoption and implementation of nutrition-based fiscal policies in India using a multi-method qualitative approach, combining a scoping review and key informant interviews. A policy triangle framework was used to identify the context, content, process, and actors affecting policy uptake. Our findings reveal a highly fragmented and contested policy space, shaped by competing commercial, political, and public health interests. Current policies focus narrowly on addressing undernutrition and tend to exacerbate overnutrition. Aggressive industry marketing of processed foods, cultural dietary preferences, and socioeconomic inequities intersect with regulatory gaps, opaque fiscal frameworks, and a fragmented political landscape (inter-ministerial conflict, industry lobbying, and limited policy focus) to hinder policy progress.

India’s current tax structure nominally taxes processed foods, but definitional ambiguities and loopholes—such as manufacturers circumventing higher tax slabs by adding minimal fruit content—undermine policy effectiveness. Nutrition-based food subsidies remain skewed towards staple crops and sugar, with fruits, vegetables, and other nutrient-rich foods lacking fiscal support to increase consumption. Fiscal policy acceptance can be improved by earmarking health tax revenue for nutrition programmes, incentivizing healthier product innovation to lower costs, and implementing targeted food subsidies to reduce undernutrition. Advancing nutrition fiscal policy in India is as much a political and institutional challenge as a technical one, hinging on clearer, nutrition-aligned tax definitions, stronger coordination between revenue- and health-focused actors, and targeted subsidies for nutritious foods.

Key messages

- Nutrition-based taxes and subsidies in India occupy a fragmented, contested policy space where revenue-focused institutions (Ministry of Finance, Goods and Services Tax Council) hold greater sway than health-focused actors, and competing stakeholder interests stall progress.
- Existing fiscal measures are undercut by design: processed foods escape higher tax slabs through definitional loopholes, while subsidies remain skewed towards staples and sugar rather than nutrient-rich foods.
- Advancing these policies is as much a political and institutional challenge as a technical one, requiring clearer, nutrition-aligned tax definitions, stronger cross-sector coordination, and proactive engagement with concerns over regressivity, farmer livelihoods, and administrative feasibility.

## Introduction

Low- and middle-income countries (LMICs) are undergoing a nutritional transition, marked by increased consumption of sugary, fatty, and highly processed foods, which collectively contribute to higher levels of obesity and diet-related non-communicable diseases (NCDs) (Popkin 2015, 2014, Popkin and Ng 2022). This health burden coexists with persistent undernutrition (Wells et al. 2020). Compounding the nutritional crisis is a shift towards sedentary lifestyles and physical inactivity (Popkin and Ng 2022). NCDs accounted for 75% of non-pandemic-related global deaths (∼43 million) in 2021 (United Nations 2021, World Health Organization 2023a).

India alone contributed 14.5% of global NCD deaths in 2019 (Ministry of Health and Family Welfare 2022, Shrivastav et al. 2023). NCDs also account for a growing share of overall health loss within India: the share of total disability-adjusted life years attributable to NCDs increased from 27% in 1990 to 63% in 2023, reflecting the rising burden of both premature mortality and years lived with disability (Pradhan et al. 2025). In 2019, 55% of NCD deaths in India occurred before the age of 70 years, compared with 41% globally (World Health Organisation 2026).

The predominant policy response to the crisis emphasizes treatment rather than prevention, even as evidence indicates that interventions targeting risk factors are highly cost-effective (World Health Organization 2017). Studies in LMICs have shown that interventions including lifestyle-, screening- and risk management-based programmes, had an incremental cost-effectiveness ratio of USD 50, USD 1574, and USD 4907 per quality-adjusted life year gained for diabetes, cardiovascular diseases, and hypertension, respectively, which were well within cost-effectiveness thresholds (Sathish et al. 2020, Zhang et al. 2021, Win et al. 2024). Among these, fiscal instruments, particularly taxes and subsidies, have significant scope to improve nutrition and health outcomes but remain underutilized globally, especially in LMICs, where implementation is often constrained by institutional and political factors (World Health Organization 2017, The Task Force on Fiscal Policy for Health 2024).

Taxes have been used historically to discourage consumption of tobacco and alcohol (Wright et al. 2017, Thow et al. 2018) and more recently to reduce consumption of sugar-sweetened beverages (SSBs), foods high in fat, sugar and salt (HFSS), saturated, hydrogenated and *trans* fats, and other unhealthy foods (Crosbie et al. 2022, World Health Organization 2023b, Pineda et al. 2024). When well-designed and effectively implemented, these taxes have the potential to generate positive health and economic effects such as reductions in morbidity and premature mortality alongside increased government revenues (Summan et al. 2020). However, adoption, efficient design, and enforcement of taxes are often constrained by political and economic challenges, including industry lobbying, lack of political will, and suboptimal policy design (Bridge et al. 2020), which in turn are influenced by local context, institutions, norms, and frameworks. Therefore, research on bottlenecks preventing implementation of country-level health taxes is important (Isaranuwatchai et al. 2020).

Fiscal measures targeting unhealthy food consumption in India remain limited—the most notable tax is on SSBs, which attract the highest Goods and Services Tax (GST) slab of 40% following the 2025 GST reform that consolidated the earlier 28% rate and 12% compensation cess into a single rate (Ministry of Finance 2026). However, other unhealthy foods remain untaxed. A modelling study found that a 10%–30% additional health tax could reduce demand by 7%–30% for SSBs and 5%–24% for HFSS foods (Varghese et al. 2024). It projected that increasing the total tax on bulk sugar purchases by confectionery manufacturers to 48% through an additional 20% health tax could decrease sugar demand by 13%–18%, while also generating substantial government revenue (Varghese et al. 2024).

There is little information on how contextual factors influence evidence-based health policymaking in India, particularly for novel fiscal tools such as health taxes and nutrition-based food subsidies (Mirzoev et al. 2014). We conducted an analytical country case study to assess how political-economy factors affect the design, adoption, and implementation of nutrition-based fiscal policies in India. The insights gathered inform a situation analysis and provide policy recommendations to strengthen health-related fiscal measures in India, with potential for applicability to other LMICs.

## Methods

We employed a multi-method qualitative approach: (i) a scoping review to identify the contextual factors that influence fiscal policy uptake and (ii) key informant interviews (KIIs) of stakeholder groups. We focused on the broader spectrum of health taxes, encompassing SSBs, HFSS foods, and junk food, alongside related subsidies on healthier alternatives (such as fruits, vegetables, pulses, and cereals). Additionally, we explored subsidies for harmful commodities, namely sugar.

The findings were mapped to Walt and Gilson’s health policy triangle (HPT) framework, which has been used to analyse fiscal policies related to NCDs (Elliott et al. 2022, Sing et al. 2023). The framework was selected as it moves beyond a narrow focus on policy content to examine how actors, interests, and institutional processes shape policy design and implementation—core concerns in political economy analysis, particularly in LMIC contexts (Walt and Gilson 1994). It considers the context within which the policy is developed, the content of reform, the processes conditional on designing and implementing it, and the actors involved influencing the policy (Walt and Gilson 1994). By explicitly incorporating these four dimensions, it enables a structured analysis of why policies are adopted, modified, or resisted in practice (Walt and Gilson 1994).

### Scoping review

**Figure 1:**
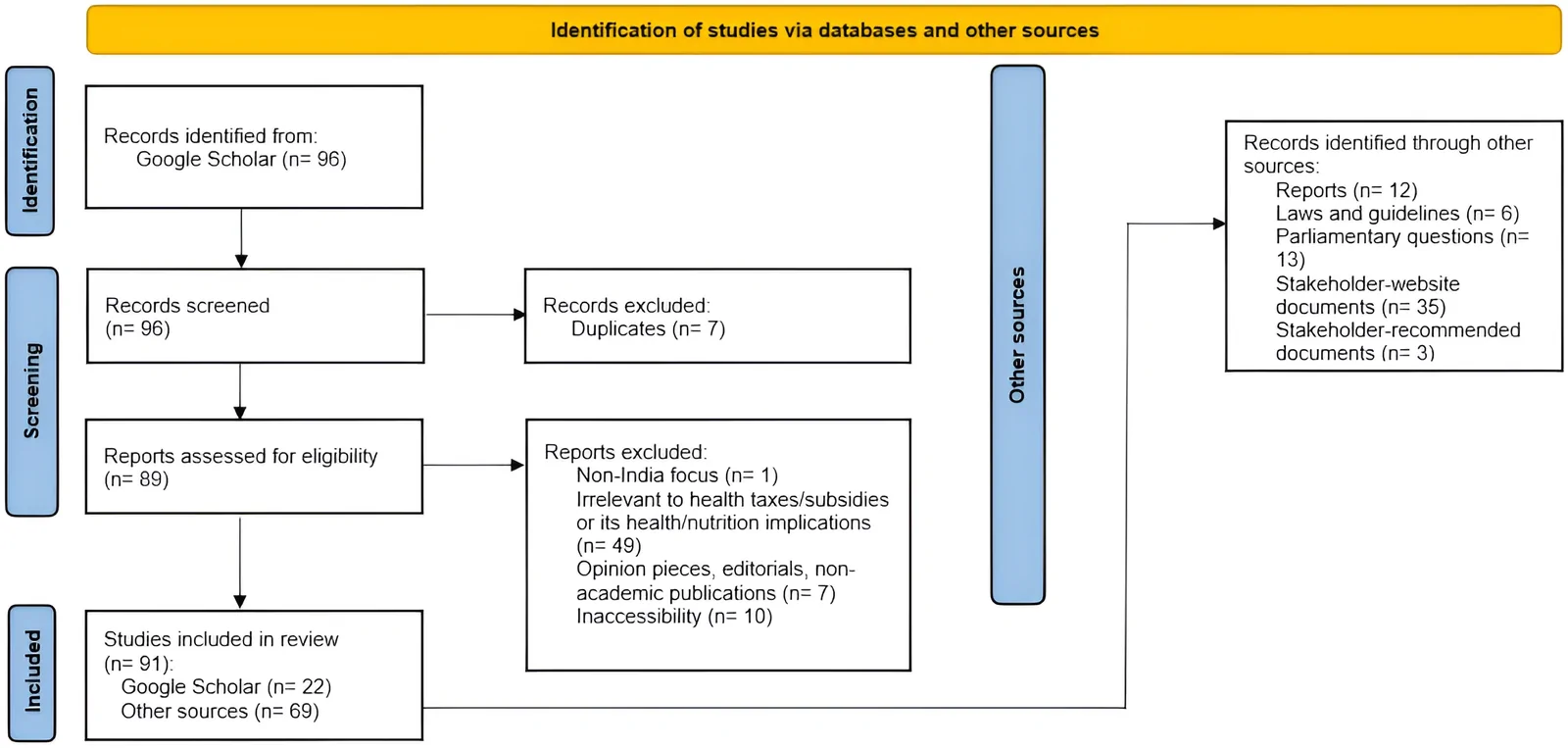
PRISMA flow diagram.

The scoping review combined a structured Google Scholar search with targeted searches of stakeholder and organizational websites for grey literature and policy documents. The Google Scholar search used broad terms related to India, food systems, and dietary risk factors, such as ultra-processed foods, HFSS foods, and SSBs, and fiscal policy instruments, including taxation, levies, cess, subsidies, and GST. The detailed search string is provided in supplementary Appendix 1 (see online supplementary material). The review followed the Preferred Reporting Items for Systematic Reviews and Meta-Analyses extension for Scoping Reviews (PRISMA-ScR) guidelines (Fig. 1) (Tricco et al. 2018). Given the interdisciplinary and policy-oriented scope of this study, Google Scholar was used as the primary academic search platform because it captures literature across public health, health economics, nutrition, agriculture, and policy domains, including some scholarly grey literature such as working papers, technical reports, theses, and preprints.

Publications from 2014 to 2024 were considered. The initial Google Scholar search yielded 96 results, of which 89 were screened based on titles and abstracts for relevance after removing duplicates (see supplementary Appendix 1). This timeframe captures critical policy milestones, including the 2015 GST rate structure report, the 2016 fat tax in the state of Kerala, and GST implementation in 2017. Studies meeting the inclusion criteria were further subjected to full-text review by the study team. The inclusion criteria for academic studies were as follows: qualitative or quantitative studies specific to the Indian setting; studies focusing on nutrition-based taxes or subsidies; and studies examining the impact of these fiscal policies on public health outcomes, healthcare expenditures, or fiscal revenue. Non-academic sources, including reports, laws or guidelines, parliamentary questions, policy documents, and stakeholder-website documents, were included where they provided relevant policy, institutional, or implementation context.

Articles focusing on alcohol or tobacco, articles that lacked relevance to health taxes or food subsidies and their health or nutrition implications in India, and opinion pieces were excluded from the review. For included studies and documents, we extracted information on the fiscal instrument or policy area examined, the relevant food or beverage category, the main policy design or implementation issues identified, and the corresponding domain of the HPT (supplementary Appendix 2, see online supplementary material). A PRISMA flow diagram (Fig. 1) summarizes the study selection process, detailing the number and type of records identified, screened, and excluded at each stage (Tricco et al. 2018, Page et al. 2021).

We also searched KI organizational websites and additional stakeholder websites for relevant grey literature, including reports, laws or guidelines, parliamentary questions, and policy documents. These stakeholders included: (i) government ministries [Ministry of Health and Family Welfare (MoHFW); Food Safety and Standards Authority of India (FSSAI); Ministry of Consumer Affairs, Food and Public Distribution (MoCAFPD); Ministry of Women and Child Development (MoWCD); Ministry of Food Processing Industries (MoFPI); Indian Council of Medical Research—National Institute of Nutrition (ICMR-NIN); Indian Council for Research on International Economic Relations (ICRIER)], (ii) think tanks, civil society organizations (CSOs) [Breastfeeding Promotion Network of India (BPNI); Nutrition Advocacy in Public Interest (NAPi); Public Health Foundation of India (PHFI); Uday Foundation; Indian Academy of Pediatrics (IAP)], (iii) industry associations [Indian Sugar Mills Association (ISMA); National Federation of Co-operative Sugar Factories Ltd]; and (iv) international organizations [World Health Organization (WHO); United Nations Children’s Fund (UNICEF); Food and Agriculture Organization (FAO)].

### Stakeholder interviews

First, we identified key stakeholder groups that may affect fiscal health policy implementation based on previous research analysing political economy factors affecting policy uptake (Ruhara et al. 2021, Dry and Baker 2022, Ahsan et al. 2023, Carriedo et al. 2023, Elliott et al. 2023, Mirza and Munir 2023, Zuleta et al. 2023). These groups are outlined in Table 1. Then we searched for KIs within these groups—which were primarily based in Delhi, the political capital of India. KIs were further identified based on academic and grey literature. Subsequently, additional KIs were identified using a snowball sampling method. This approach was employed given a limited number of experts specializing in fiscal policies for nutrition. Between February 2024 and September 2024, a total of 18 semi-structured KIIs were conducted by A.S. and D.S., either in person (*n* = 4) or online (*n* = 14). The duration of the interviews was 40 to 60 min. Each interviewee was assigned a neutral alphanumeric code (e.g. KII-01) to attribute quotations while preserving anonymity and indicating repeated quotes from the same participant.

**Table 1:** List of stakeholder interviews by groups.

Stakeholder group	Number of interviews
Academia/researchers	4
Think tanks/CSOs/NGOs/consultancies^a^	4
Consumer interest sector	1
Food and beverage industry representatives and industry consortiums	2
Government	
Government (finance, health)	1
Government (executive power)	1
Government (legislative)	1
Health sector representatives	2
Media	2
Total	18

^a^NGO, non-governmental organization.

The interview guide was developed based on an analysis of relevant published literature and policy reports. The guide consisted of eight topics: (i) participants’ knowledge and understanding of fiscal policies for health, (ii) public awareness of fiscal policies and nutrition, (iii) policy-enabling environments, (iv) challenges in food and nutrition security, (v) the double burden of malnutrition in India, (vi) upscaling of nutrition-sensitive agricultural policies, (vii) collaborations with international partners, and (viii) recommendations and future perspectives to improve policy uptake. Prior to the interviews, participants were provided with written information about the study and gave written consent to participate. All interviewees consented to audio recording. To encourage open discussion, interviewees were assured of anonymity, and that no personal identifiers would be recorded. The number of interviews conducted per stakeholder group is given in Table 1.

### Data analysis

Stakeholder interviews were transcribed verbatim using Microsoft Word’s Transcription feature by D.S., S.D., and one research assistant (RA). The transcripts were reviewed twice—first by S.D. and an RA and then by D.S. A codebook was prepared based on the interview guide and the first screening of the interview transcripts. The interviews were then analysed using NVivo (version 14). After the data extraction, the codebook was refined to align with the primary themes of the study by grouping similar codes into hierarchical subthemes. A thematic analysis was conducted using a hybrid deductive–inductive approach. Deductive codes were derived from the four dimensions of the HPT framework (context, content, process, actors); inductive coding was used to capture emergent themes not anticipated by the framework. Codes were iteratively refined and grouped into the analytical themes presented in the Results section.

### Patient and public involvement

No members of the public or patients were involved in research design, analysis, or dissemination.

## Results

We identified key areas of focus under each domain of the policy triangle by synthesising relevant literature and integrating stakeholder perspectives. The scoping review identified three dominant areas of evidence. First, existing academic literature primarily focuses on the design and potential effectiveness of fiscal measures, particularly taxes on SSBs, with relatively limited attention to subsidies. Second, several studies highlight implementation challenges, including definitional ambiguities, regulatory gaps, and institutional fragmentation. Third, political economy factors, such as industry influence, competing government priorities, and limited public awareness, emerge as critical barriers to policy adoption. These findings informed the thematic structure of the analysis and were complemented by stakeholder interviews. Table 2 summarizes the domain description and key themes that emerged during our analysis.

**Table 2:** Identified themes under the policy triangle framework.

Domain	Description^a^	Identified themes
Context	Political, social, cultural, economic, and international factors within which actors operate and policies are formulated	Cultural factors in Indian diets Nutrition-related issues and the triple burden of disease Market structure of food and beverages sector Role of marketing Public awareness
Content	Technicalities or design of the policy	Policy design and framing Vulnerable consumer groups Alternative policy measures
Process	Activities that shape the identification of issues and the formulation, negotiation, communication, implementation, and evaluation of policies	Political considerations Industrial lobbying Media influence Challenges in agricultural and food subsidies
Actors	Individuals, groups, and organizations that engage with and shape policy decisions	Stakeholder groups—existing, potential, and conflicting responsibilities

^*a*^Descriptions taken from Walt and Gilson (1994).

### Context

#### Cultural factors in local diets

Indian dietary patterns are heavily influenced by both traditional practices and modern shifts. The scoping review indicates a growing shift towards ultra-processed food consumption in the country, driven by urbanization. This trend was also reflected in stakeholder interviews, where KIs noticed an increasing presence of foods traditionally popular in Western countries in Indian urban areas and a growing preference for imported ‘superfoods’—foods high in nutritional density (e.g. blueberries and raspberries)—despite the availability of locally produced alternatives such as amla, guava, and green leafy vegetables. Nonetheless, traditional diets remain dominant due to cultural and familial influences. Importantly, KIs emphasized that nutrition challenges should not be framed as a binary between Indian and Western foods:

There are a lot of Indian junk foods… Western foods are very heavy on salads, and those things also has a lot of good things, right?… So, I don’t think that this is Western versus Indian life. (KII-01)

Studies have highlighted such policy biases in Kerala’s fat tax, which targeted ‘branded’ fast food chains, including multinational chains like McDonald's, KFC and Domino's, while excluding fried and sugary Indian foods (Singh et al. 2019).

Although industry stakeholders argued that the per capita consumption of SSBs and HFSS foods remains relatively low in India, comparative data suggests a more nuanced picture. The annual per capita sales of processed foods in India almost doubled, rising from USD 31.3 in 2012 to USD 57.7 in 2018 (WHO Country Office for India 2023). During the same period, India’s annual per capita consumption of sugar rose modestly from 18.1 kg to 19.5 kg, still below the global average of 22.6 kg (Indian Sugar Mills Association 2019). Additionally, in 2018, Indian adults reported one of the lowest average intakes of SSBs globally at 0.2 servings per week, compared to a regional average of 0.7 servings per week in South Asia (Lara-Castor et al. 2023).

While these figures suggest current low consumption levels, they obscure critical trends and disparities. Findings from our scoping review revealed that consumption of ultra-processed foods is likely to grow by at least 6.84% by 2032, signalling an impending obesity pandemic in India (WHO Country Office for India 2023). Moreover, low national per capita figures mask varying consumption patterns across geographical and demographic groups within and between states (ICMR-National Institute of Nutrition 2019, Mukherjee et al. 2022). Several KIs also identified three key distinctions between local dietary preferences and global standards, underscoring the need for a localized approach to nutrition policy: (i) perceptions that India’s hotter climate may increase salt needs because of greater sweating and electrolyte loss, (ii) heightened NCD risk observed within South Asian populations, and (iii) regional dietary heterogeneity. Compounding these factors, a government stakeholder stated that cultural barriers may impede implementation of taxes; e.g. a salt tax may evoke memories of the ‘Salt Satyagraha’ or Salt March during India’s independence struggle.

#### Nutrition-related issues and the triple burden of disease

Nearly all KIs emphasized India’s triple burden of malnutrition, characterized by high levels of undernutrition, rising rates of obesity and chronic diseases, and significant micronutrient deficiencies, or ‘hidden hunger’. Specifically, academics described both undernutrition and overnutrition as social and commercial issues that continue to be misunderstood as medical problems. They criticized the focus of Indian policies on undernutrition, with little attention paid to micronutrient deficiencies and overnutrition, previously considered a ‘rich man’s illness’.

They noted how NCDs such as type 2 diabetes, hypertension, chronic kidney disease, and fatty liver disease are increasing due to novel nutrition factors. One KI illustrated how dietary habits, independent of alcohol consumption, contribute to liver disease:

Even a person who’s not having alcohol, but is having the wrong kind of diet, can get fatty liver, and that’s not manifested until an extreme stage. (KII-11)

#### Market structure of food and beverages sector

Evidence from the scoping review highlights that India’s food and beverage sector remains largely unorganized, with a growing but fragmented processed and ultra-processed food industry. In 2021, 80% of production and 70% of sales of ultra-processed foods were through independent, small local grocers (*kirana* stores), even as e-commerce sales channels grew modestly by 1.75% between 2019 and 2021 (WHO Country Office for India 2023). This growth was driven by beverages, which dominated the market by retail sales volume, followed by chocolate and sugar confectionery, ready-made and convenience food, and salty snacks (WHO Country Office for India 2023). Similarly, the non-alcoholic beverage sector grew at a rate of 14.5% in terms of total sales volume between 2010 and 2019, with 80% of the market operating informally, consisting of small, unregistered businesses operating without formal regulatory oversight (Mukherjee et al. 2022).

The widespread informality and decentralized nature of production in the sector create a complex landscape for taxation. Academics and government KIs cautioned that these characteristics undermine tax effectiveness by making revenue collection unpredictable. They noted that limited regulatory oversight lowers compliance and may push more businesses into the informal economy, especially as firms seek to avoid higher tax burdens.

#### Role of marketing

Marketing strategies, including product design and placement, pricing, and promotion, have increased demand for processed foods across income groups. Health sector stakeholders asserted that processed foods are deliberately formulated to have addictive qualities, combining fat, salt, and starch to activate the brain’s reward system. Contributing to health concerns, most sugar produced in India is plantation white sugar, refined through the double sulfitation process, which involves addition of sulfur and is considered to be more harmful than raw sugar (KPMG India 2007, Central Pollution Control Board 2018). The industry stakeholder countered that sugar is an affordable energy source:

Anything in excess is bad, not only sugar… sugar is the cheapest source of energy. (KII-08)

A CSO stakeholder expressed concerns regarding the targeted marketing strategies of multinational companies and Indian conglomerates, which employ a ‘bottom of the pyramid’ approach to reach low-income consumers with smaller, affordable processed food packaging. Examples include 100 ml cartons of sugar-sweetened juices and single-rupee packets of leftover, broken pieces of chips. Simultaneously, high-income consumers drive the demand for premium processed foods and restaurant dining. This dual-pronged strategy, coupled with lifestyle changes such as time constraints faced by working women, has accelerated the shift from homemade meals to pre-packaged and restaurant foods across both rural and semi-urban areas (Popkin and Ng 2022).

Companies also use product placement and advertising tactics to ‘manufacture demand for a product’. For example, a health sector representative pointed out how chips are often placed at children’s eye levels in stores to drive impulse purchases. KIs from media and CSO groups noted that despite regulations prohibiting advertising unhealthy foods to children, these products are frequently marketed during family television programmes. Companies avoid labelling their products as HFSS, instead marketing them as ‘indulgent treats’, thereby evading transparency about their potential health risks. Misleading advertising, including celebrity endorsements, ‘junk science’, or unsubstantiated health claims, such as those of red wine or refined oils, further confuses consumers.

Government and health sector KIs added that advertisements and peer groups may promote processed products as symbols of status and modernity. The problem, they argue, is largely market-driven: substitutes for breast milk are manufactured, advertised, and promoted by medical professionals and other influential figures. Furthermore, use amongst higher-income women may affect domestic workers, both of whom may persuade new mothers that these products are beneficial for infants. However, families who are unable to afford adequate quantities of such substitutes often resort to dilution, leading to diarrhoea and malnutrition among infants. Similarly, usage of biscuits as ‘complementary foods’ for children, despite their limited nutritional value, and the promotion of immunity-boosting products following the COVID-19 pandemic, were other cited examples.

#### Public awareness

Public awareness of nutrition in India remains concentrated within niche groups and is hindered by persistent misconceptions and lack of organized advocacy. Positive steps are the regular release of dietary guidelines by national authorities and advocacy groups. Another encouraging trend may be the growing recognition of sugar’s health risks:

And those people who are not consuming it [sugar sweetened beverages], or say Diet Cola instead, are doing it because they’ve heard that sugar is bad. So, the awareness is there. It’s not because that’s expensive or that’s cheap or whatever, they’re both the same price. (KII-11)

A government stakeholder suggested simplifying public communication by focusing on single nutrients, such as sugar in beverages, salt in savoury items, and fat in fried foods. Health sector representatives praised the FSSAI’s *Aaj Se Thoda Kam* (translates to ‘from today, a bit less’) campaign for promoting gradual reductions of sugar, salt, and unhealthy fat in diets.

The use of ‘nudges’ alongside labelling norms may also influence consumer purchasing decisions. In Bihar, a study found that a brief, point-of-sale script increased demand for a fortified food product and reduced price sensitivity by 18% among women who had previously undergone a behaviour change and communication intervention, but had no effect on those without prior exposure (Dizon and Yu 2021). As consumer awareness grows, firms are adjusting their products to meet these evolving expectations, with industry KIs acknowledging that they will eventually have to comply with market demand.

These shifts in public awareness and purchasing behaviour can enhance the impact of taxes on harmful foods or subsidies for nutritious alternatives by increasing consumer responsiveness and political feasibility. Both mass awareness campaigns, such as those led by the FSSAI, and targeted nudges like the point-of-sale messaging seen in the Bihar study, can help shape consumer preferences and reinforce the goals of the fiscal policy. Without complementary efforts in awareness-building and communication, such fiscal policies may face resistance, be misunderstood, or fail to shift demand meaningfully.

### Content

#### Policy design and framing

The Ministry of Finance outlined the logic behind current food tax rates during the 2017 GST reforms; the intention was to exempt unprocessed food sold directly by farmers, reducing the tax burden on low-income households (Poddar and Ahmad 2009). Accordingly, essential items such as fresh fruits, vegetables, and cereals have remained exempt or minimally taxed, while sweetened carbonated and caffeinated beverages have been classified as demerit goods and taxed at the highest rate, signalling a shift towards public health considerations. India’s 2025 GST reform reinforced this orientation: it consolidated the tax slabs and withdrew the compensation cess, leaving most staple and unprocessed foods untaxed and packaged or processed foods taxed at a low rate, while sweetened carbonated, aerated, and caffeinated beverages were moved to the highest (40%) slab. Another key consideration was to maintain revenue neutrality by avoiding excessive exemptions that might necessitate higher taxes on other essential goods such as medicines or fuel (Poddar and Ahmad 2009). These historical tax policies continue to inform ongoing debates on effectively taxing processed foods, while navigating affordability, equity, and public health objectives.

Fiscal policies for health and nutrition in India have been criticised for lack of transparency and consistency (Drèze and Khera 2015, Standing Committee on Food, Consumer Affairs and Public Distribution 2017). Frequent budget reporting changes that hinder year-to-year comparability, coupled with a lack of access to segregated, scheme-specific data, suggest potential intentional obfuscation. A government stakeholder criticized the tax authorities as being complacent, resistant to innovation, and ‘comfortable’ with the existing system’s revenue generation. Further pressures from the World Trade Organization create a fundamental conflict between opening markets to large food corporations and maintaining food security for the local population.

Specifically, for a health tax, defining ultra-processed foods and distinguishing between food processing levels has been a complex process, with differing preferences among key stakeholders, including the FSSAI, ICMR-NIN, the GST Council, and leading advocacy groups (BPNI and NAPi). The FSSAI classifies food as healthy or unhealthy, creating confusion with GST tax classifications of sin and demerit goods. The WHO recommends labelling free sugar, but FSSAI mandates ‘added’ and ‘total’ sugar declarations. According to a government stakeholder, the GST Council avoids detailed label analysis and prefers using total sugar as the basis for taxation, citing limited expertise to distinguish added sugar on product labels.

On earmarking such tax revenue, KIs agreed that directing it to public health initiatives could help garner public support, given the fungibility of government revenue. However, a health finance official stated:

You can’t have too many objectives thrust into tax policy, the tax policy is supposed to raise revenue and that’s it… and that has to be done with a minimum impact on resource allocation or resource distortions, minimizing the compliance cost, and minimizing the administrative cost. (KII-04)

Similarly, in the case of food subsidies, a KI contended that the term ‘subsidy’ misrepresents the nature of social sector spending, suggesting instead that such expenditures should be understood as the state’s legitimate allocation of public resources to advance societal welfare, rather than as a mere financial concession. Several KIs noted that gaining farmer support for such reforms may require compensatory measures, as taxes on sugar-intensive foods could reduce demand for crops such as sugarcane, while nutrition-based food subsidies involve trade-offs between supporting subsistence crops that improve nutrition and cash crops that increase income.

### Process

#### Political considerations

India’s nutrition and health policymaking is also influenced by political dynamics. Frequent changes in ministry leadership disrupt policy continuity, while internal conflicts between government ministries, each with different key performance indicators or targets, hinder the development of coherent nutrition policies. For example, implementation of front-of-package labelling was partly delayed due to lobbying efforts and conflicting objectives between the MoCAFPD, the MoHFW along with the FSSAI (Gupta et al. 2023). Political backing of the POSHAN Abhiyan as India’s flagship programme for improving nutritional outcomes and the recent promotion of millets as nutrient-rich staples were also cited as key examples of how state support selectively prioritizes certain nutrition interventions.

Our scoping review of parliamentary questions revealed that although the opposition has raised valid concerns about the health impacts of junk and ultra-processed food consumption, particularly among children, the scope of its criticism has been relatively narrow. Several questions overlapped, often inquiring about the government’s awareness of the rise in junk food consumption and its link to chronic diseases, as well as policies on advertising regulations and labelling norms. The emphasis was heavily skewed towards school-based interventions, with limited attention to systemic changes such as supply-side reforms, fiscal incentives for healthy food, or long-term strategies in nutrition education. When questioned about a possible ‘junk food tax’, the government emphasized its reliance on FSSAI’s labelling norms ‘to enable consumers to make an informed choice’ (Government of India 2023). Additionally, a government stakeholder criticized bureaucratic inertia, observing that some officials prioritize securing high profile or ‘plump’ ministries to advance their political standing rather than drive policy reforms.

One CSO stakeholder emphasized that the direction of fiscal policies particularly related to subsidies is often skewed by the overarching economic focus on industrialization, with the government’s fiscal agenda supporting industries through ‘a kind of hidden, larger system of subsidy’. The scoping review analysis revealed that similar incentives, such as full or partial tax exemptions, import duty waivers for export-oriented units, and 100% foreign direct investment via the automatic route allowing foreign investments in Indian companies without government approval have facilitated growth in sectors like biscuits, chocolates, and ice creams (Krishnamoorthy et al. 2020). Subsidies for seemingly healthy foods can also be counterproductive if industries use subsidized products as raw materials to produce unhealthy ones (e.g. millet subsidy used for producing millet chips). A stakeholder proposed integration of reducing harmful nutrient content into the existing production-linked incentive scheme to incentivize innovation, research, and development.

In the case of sugar, the MoCAFPD tasked with regulating sugar’s availability and cost, finds itself balancing consumer health with economic interests through three pathways. First, an industry representative explained that, when sugar prices fall, the ministry often promotes sugar consumption to stabilize the market. Second, farmer incomes also depend on sugar as a raw material being supplied to the non-alcoholic beverage industry (Mukherjee et al. 2022). Third, an industry stakeholder remarked upon India’s distinct advantage of quality, freight, and time in sugar exports, necessitating bolstering of domestic sugar production to meet the country’s export potential.

#### Industry lobbying

The junk food and sugary beverage industries exert significant influence on policymakers through their contributions to political funding and their role in job creation. One interviewee highlighted that reducing the production of processed food items could directly affect farmer incomes, emphasizing the economic dependencies involved. Consequently, in some cases, imposing high taxes on these industries could shift the financial burden to raw material suppliers, including farmers and supply chain partners (Mukherjee et al. 2022). A media stakeholder cautioned against related political backlash:

We don’t want to antagonize the sugar cane lobby, especially the farmer lobby and set a war into that. (KII-06)

Alternatively, industries might absorb the costs through reduced profit margins instead of raising consumer prices. Encouragingly, KIs noted that there is considerable consensus in India, even among industries, on implementing a tiered sugar tax, offering a possible resolution. This consensus emerged from the understanding that a tiered approach could balance public health objectives with industry concerns by targeting products based on their sugar content, while also allowing industries to adapt gradually to the new regulations.

#### Media influence

Media plays a critical role in shaping public perceptions and discourse of health programmes. An informant referenced media reports on the Anemia Mukt Bharat Program (Ministry of Health and Family Welfare 2024), where coverage of the side effects of iron supplementation triggered public fear. Furthermore, corporate interests may influence media coverage, as one informant stated:

Media may report, but they sanitize it so much so that they do not hurt the interests of their corporations or friendly government. (KII-03)

This influence extends to political and fiscal entanglements, with corporate interests shaping media coverage to downplay resistance movements against harmful foods. Similarly, government endorsements, as seen with the recent popularity of millets and turmeric, demonstrate how political engagement shapes consumer perception and influences product choices in India. Believing ‘government reacts to media and publicity’, a KI recommended publishing multiple reports from diverse stakeholders to ‘generate visibility’ and prompt policy action on processed food consumption.

#### Challenges in agricultural and food subsidies

Interview discussions focused on subsidies for sugar and healthy foods like fruits, vegetables, cereals, and pulses. Academic and media stakeholders criticized agricultural subsidies for primarily benefiting large corporations rather than small farmers, pushing the sector towards industrial farming and livestock rearing. A stakeholder, with a background as a sugar farmer and serving in a leadership role within the industry association, emphasized that cash crops like sugarcane remain favoured by farmers due to fixed prices and less market uncertainty:

I grow sugarcane simply for three reasons. It is protected from both sides. My raw material price is fixed under a statute FRP—Fair and Remunerative Prices. My raw material, when it is ready, I don’t have to run around for marketing… the sugarcane pricing is covered under the statute of Essential Commodities Act, so as a farmer, it’s the most farmer-friendly crop. (KII-08)

Civil resistance movements, such as protests against Coca-Cola’s disproportionate water usage in Plachimada, Kerala, and Mehdiganj, Uttar Pradesh, are gaining traction in response to these practices. Technological trends like direct farmer-to-consumer e-commerce, aim to reduce dependency on middlemen.

Government efforts to improve food subsidy delivery in India have focused on Direct Benefit Transfer (DBT), Aadhaar-enabled Payment Systems, and the One Nation One Ration Card (ONORC) scheme. DBT, launched in 2015, transfers welfare benefits directly to bank accounts, but faces uneven regional adoption (Chakrabarty 2023, Tiwari and Kamila 2023). ONORC, introduced in 2019, enables eligible beneficiary migrants to access food rations from any Fair Price Shop in the country (Dalberg 2022). Previously, eligibility for food rations was limited to the region in which the ration card was issued, whereas with ONORC, one can use the ration card to access food grains in any part of the country. Despite progress in improving food subsidy delivery mechanisms in India, issues like Targeted Public Distribution System leakages, grain diversion, poor foodgrain quality, and targeting errors persist, worsened by reliance on the outdated 2011 census data (Desai et al. 2016, Panigrahi and Pathak 2018, Roy 2021, Supreme Court of India 2022).

Additionally, systemic issues in the design of subsidies remain unresolved. For instance, periodic revisions in the central issue price, which is the government-fixed price at which foodgrains are made available for beneficiaries of the National Food Security Act, have not occurred since 2002, despite increases in the minimum support price (MSP), which have escalated food subsidy expenditures (Standing Committee on Food Consumer Affairs and Public Distribution 2017, Chand and Singh 2023). This is largely due to political and fiscal complexities. In several states, where retail prices are aligned with or lower than the central issue price (e.g. Karnataka, West Bengal), and include free distribution in some cases (e.g. Tamil Nadu), any increase would shift the financial burden onto already resource-constrained state governments. Besides, subsidized food pricing is often tied to electoral strategies. Expanding subsidies onto other foods would also add pressure on the ballooning food subsidy bill and raise concerns about who bears the financial burden—government, farmers, or intermediaries (Venkatraja 2016). Lastly, perishable healthy foods like fruits and vegetables would require more intricate food distribution systems than staple grains and cereals.

### Actors

To highlight the complex interplay of varied stakeholder interests, Table 3 lists examples of existing responsibilities, potential contributions, and conflicts of key stakeholders involved in India’s nutrition and health policies, identified through interviews and our scoping review. The existing responsibilities of stakeholders often lead to conflicts and challenges, such as funding limitations for academia, lobbying by industries, and competing priorities among government departments. Potential responsibilities suggest ways stakeholders can enhance their impact, e.g. by translating research into actionable policies or adopting sustainable industry practices. The fragmented and informal structure of India’s food and beverage sector limits regulatory oversight and weakens uniform implementation of fiscal measures. This decentralization diffuses accountability across various actors, such as small or unregistered enterprises and independent retailers, making compliance difficult to monitor and shifting policy influence towards larger, organized industry players who are better positioned to engage in lobbying and policy negotiation.

**Table 3:** Examples of existing, potential, and conflicting responsibilities of stakeholders.

Stakeholder group	Existing responsibilities	Responsibilities to expand/develop	Challenges
Academia/researchers	Provide evidence on malnutrition, anaemia, and other health issues. Conduct studies on fiscal policy for health. Support health programmes with data.	Translate research into actionable policy briefs. Develop broader cost–benefit analyses. Collaborate with advocacy groups.	Limited funding for independent research. Pressure to focus on tax-related data collection. Restricted roles in international collaborations.
CSOs/advocacy groups (e.g. BPNI, NAPi)	Raise concerns about nutritional discrepancies. File complaints to authorities. Act as a watchdog for government policies.	Advocate for higher taxes on unhealthy foods. Form opposition to tax reductions on unhealthy products. Lead awareness campaigns.	Struggle to compete with corporate lobbying. Limited resources for large-scale campaigns.
International bodies (e.g. WHO)	Advocate for international health strategies. Support research and benchmarking. Promote food fortification initiatives.	Collaborate with Indian policymakers to encourage region- and country-specific guidelines for nutrient thresholds.	Conflicting sugar taxation recommendations. Limited industry engagement despite need for local support.
FSSAI	Regulate nutritional labelling. Oversee fortification programmes. Engage with industry to ensure compliance.	Develop stricter regulations on marketing and advertising. Improve representation in advisory committees.	Inconsistencies in food classification. Lack of alignment with WHO’s sugar categorization standards.
Ministry of Finance	Conduct pre-budget consultations. Refine tax policies. Increase revenue while minimizing economic distortions.	Align fiscal policies with health goals. Engage with state-level stakeholders on regional impacts.	Balancing economic priorities with health-focused taxation. Conflicting tax objectives.
GST Council	Tax products based on FSSAI labels. Refine GST system to ensure equitable tax distribution.	Collaborate with nutritionists and state health departments. Sensitize GST members on health impacts.	Challenges in balancing affordability with taxing unhealthy products. Lack of detailed sugar breakdowns.
MoHFW	Lead health programmes like Anemia Mukt Bharat and Nutrition Rehabilitation Centres for severe and acute malnutrition. Provide dietary guidelines and advocate for reducing HFSS consumption.	Frame health taxes as important public health tools. Advocate stronger regulations on food marketing.	Navigating competing government key performance indicators for industry growth versus health policies.
Ministry of Women and Child Development	Provide health and nutrition through services such as Anganwadi, Poshan Abhiyan, and take-home ration schemes. Collaborate with MoHFW on child nutrition.	Use technology to enhance real-time nutrition monitoring. Improve programme efficiency.	Data integration challenges with other departments. Fragmented oversight.
Industry groups/associations	Increase industry profits. Lobby against restrictive health policies like Front-of-Package Labelling.	Reformulate products to align with health standards. Adopt sustainable practices.	Opposition to health-focused regulations. Prioritizing profitability over public health.

Fiscal policy ownership is concentrated within the Ministry of Finance and the GST Council, which determine tax design and rates, while health-oriented ministries play a largely advisory role. Food and nutrition subsidies, by contrast, are governed by sectoral ministries and implemented by state governments. This division may create an imbalance, with revenue-focused institutions holding greater decision-making power than health actors.

Additionally, our analysis identified other stakeholder groups and institutions that could potentially contribute to the policymaking process for nutrition-based taxes and food subsidies (supplementary Appendix 3, see online supplementary material). These stakeholders include various government ministries (such as those overseeing agriculture, commerce, education, and food distribution), professional organizations (e.g. the Indian Society of Lifestyle Medicine, the Federation of Hotel and Restaurant Associations), and regulatory bodies (e.g. the Medical Council of India, the Telecom Regulatory Authority of India). They play a vital role in mediating food industry issues, gathering on-ground realities, and facilitating collaboration across sectors.

## Discussion

This study analysed the political economy factors influencing the design, adoption, and implementation of health taxes and nutrition-based subsidies in India, offering insights and recommendations to enhance fiscal health measures with relevance for other LMICs. Applying Walt and Gilson’s policy triangle framework (Walt and Gilson 1994), this study examined four interconnected dimensions underlining such a policy: context, content, process, and actors. Taken together, three central lessons emerge from the Indian case, organized along the content, process, and actors dimensions of the HPT, with context cutting across all three. On content, definitional ambiguities in tax design—exemplified by India’s GST classification of beverages, where firms add minimal fruit content to avoid higher slabs—substantially weaken the health intent of fiscal measures; clearer and nutrition-aligned tax definitions are essential. On process, fragmented institutional arrangements, including inconsistencies between FSSAI classifications and GST structures, and limited administrative capacity to assess sugar content, constrain implementation in a largely informal and decentralized food system; stronger inter-agency coordination and investment in administrative capacity are needed. On actors, industry lobbying (e.g. SSB sector resistance to higher rates) and public concerns about regressivity shape both adoption and design; gradual implementation, transparent use of revenues, and proactive stakeholder engagement can help build political and public support.

The nutritional landscape in India is characterized by the coexistence of undernutrition, rising NCDs, and micronutrient deficiencies. This triple burden of nutrition is shaped by cultural dietary practices and the increasing availability of ultra-processed foods. Socioeconomic inequities further exacerbate these issues, with limited awareness and accessibility hindering healthier food choices. This shift reflects broader lifestyle changes and evolving market dynamics specific to India that policies must address.

The complex nutritional challenges underscore the urgency for fiscal policy interventions. However, the effectiveness of tax policy is hindered by regulatory gaps, limited bureaucratic capacity, and inconsistent enforcement. Framing of fiscal policies also plays a pivotal role in determining public acceptance and reflects process and context elements of the policy triangle. Framing in India must account for cultural sensitivities and structural inequalities to ensure resonance with diverse stakeholders.

Additionally, effective policy design representing the content aspect of the policy triangle is paramount for the success of fiscal measures like India’s SSB tax, which has faced challenges due to definitional ambiguities and misaligned tax structures. The initial 2017 SSB tax targeted aerated beverages with no fruit content in the highest tax slab. Leveraging this, local beverage companies would add a minuscule amount of fruit content such as fruit pulp or extracts, e.g. 2.5% or 5%, to claim a 12% GST instead of 40% (Sarkar 2018, Law et al. 2021). In another definitional challenge, the FSSAI said that beverages with fruit content between 5%–10% would be called carbonated beverage with fruit juice (Sarkar 2018). Therefore, it was decided to put carbonated fruit juices into the 40% tax bracket in the 48th GST Council meeting in December 2022 (Joseph 2023); the 2025 GST reform retained this 40% treatment while removing the separate compensation cess.

This approach, based on aeration rather than sugar content, contrasts with WHO recommendations for added sugar-based taxation (Joseph 2023). Lessons from countries like Thailand demonstrate how incremental tax structures can aid enforcement. Thailand’s graded SSB excise tax, introduced in 2017, incrementally increased every 2 years, giving industries time to reformulate products and normalized the tax amongst the public (World Cancer Research Fund International 2018). This highlights the importance of policy design that anticipates and accommodates the administrative capacities of institutions and consumer behaviour.

Stakeholder interests and influence, constituting the ‘actors’ component of the policy triangle, play a pivotal role in shaping policy outcomes. In India, the food processing industry exerts significant lobbying power, often opposing fiscal policies like taxes on HFSS products. The SSB industry strongly advocates for reduced tax rates and removal of demerit goods classification (Mitra 2017, Business Standard 2023). At the same time, potential public resistance, particularly from lower-income consumers, complicates implementation, as taxes on junk foods are perceived as regressive. Addressing these challenges may require compensatory subsidies for healthier options or more equitable tax structures. The findings also suggest that market structure itself shapes the feasibility of health taxation. In fragmented and informal food systems such as India’s, administrative complexity and uneven enforcement may incentivize simpler but less nutrition-sensitive tax designs, potentially limiting the public health impact.

Framing policies with a clear revenue utilization strategy can help build stakeholder support under the content aspect of the policy triangle. In Ghana, for instance, scepticism about SSB taxation stemmed from uncertainty over how the revenue would be used (Singh et al. 2023). By contrast, earmarking tax revenue for health initiatives, such as small-scale farming or public awareness campaigns, may garner greater public acceptance. In South Africa, stakeholders viewed combining SSB taxation with support for subsistence farmers to offset potential harms while improving public perception of the policy favourably (Kaltenbrun et al. 2020). This, however, would be challenging as India does not allow for the earmarking of tax revenues, and would require additional legislation.

Political commitment and contestation among actors of the policy triangle also shape policy adoption and implementation. In Indonesia, for example, political debates over health taxes divided government institutions and led to media narratives that favoured industry arguments (Ahsan et al. 2023). Policy networks, including collaborations between academia, civil society, and international organizations, are critical to build momentum and counteract industry opposition. However, as seen in Mexico, such networks must balance advocacy with principles of governance, including transparency and accountability, to avoid undermining public trust (Carriedo et al. 2023).

Current government subsidy support varies significantly across healthier foods and their producers, including staple crops, coarse cereals and pulses, horticulture, dairy, and fisheries (Chand and Singh 2023). Rice and wheat benefit significantly from subsidies due to their higher usage of subsidized inputs such as fertilizer, irrigation water, and electricity, while higher MSPs for pulses and oilseeds help encourage their cultivation and help fulfil nutritional needs (Chand and Singh 2023). Fruits and vegetables lack price assurance schemes while the dairy sector operates through cooperatives with fixed procurement prices, relying on institutional support instead of subsidies (Chand and Singh 2023). Current efforts on enhancing efficiency and reducing leakages in the food system should be continued (Drèze and Khera 2015, Standing Committee on Food Consumer Affairs and Public Distribution 2017, Supreme Court of India 2022) as the focus becomes the subsidization of nutritious food.

Our study has important limitations. First, results from stakeholder interviews may include potential subjectivity and bias, as participants’ perspectives may reflect personal or organizational interests rather than those of the wider stakeholder group. Second, KIs in our study were primarily based in urban regions, potentially excluding rural and marginalized voices. Third, the scoping review was constrained by the availability and quality of data. The exclusion of certain grey literature, such as media articles and unpublished reports due to resource constraints, may have created gaps in the analysis. The use of Google Scholar may limit reproducibility and exclude studies specifically indexed in databases, although it allowed broader inclusion of literature.

Despite these constraints, the findings offer a potential framework through the HPT for understanding and advancing nutrition-based tax policies in India and have significant policy implications. They are framed within the context of the triple burden of malnutrition, India’s nutrition landscape shaped by cultural dietary habits, increasing ultra-processed food intake, and entrenched socioeconomic inequalities. On content, improving fiscal policy design through clearer tax definitions and integration with agriculture and food regulation suggests that phased implementation may enhance uptake. In terms of process, addressing regulatory gaps, limited institutional capacity, and inconsistent enforcement is critical. For actors, mitigating conflicts among stakeholders, ranging from competing government priorities, industry lobbying, media influence, and public resistance, can enhance policy acceptance.

The strength of this study lies in its use of diverse data sources—a scoping review combined with KIIs—which together provide a broader and more grounded view of India’s fiscal policy landscape for nutrition than either source could on its own. By integrating published evidence with the situated knowledge of policy actors, this work contributes a localized political-economy analysis of food-based health taxes and subsidies and identifies three priorities for advancing fiscal tools in similar LMIC contexts: clearer and nutrition-aligned tax design, stronger institutional coordination, and strategic stakeholder engagement to build political and public support. Building on this foundation, future research could examine sectoral coordination, state-versus-central implementation strategies, distributional effects of such policies, and participatory approaches to fiscal interventions in nutrition.

## Supplementary Material

czag093_Supplementary_Data

## Data Availability

Data may be obtained upon request.

## Reflexivity statement

Three of the four authors are based in low- and middle-income countries, reflecting diverse regional perspectives. The authorship team includes two male researchers with PhD degrees and two female graduates, ensuring a balanced representation in terms of gender and academic seniority.
