# Supplementary material for: The political economy of novel food taxes and subsidies in India: insights from stakeholder interviews and a scoping review
Source: Health Policy Plan. 2026 Jul 21;41(8):1416–28. doi: 10.1093/heapol/czag093 (PMC13586620; doi:10.1093/heapol/czag093)
Supplement: Supplementary file 1 [file czag093_supplementary_data.docx]

Supplementary Appendix

Appendix A: Google Scholar Searches Assessed for Eligibility

**Search string**
[allintitle: ("India") AND ("agriculture" OR "agricultural" OR "food" OR "nutrition" OR "fat" OR "sugar" OR "salt" OR "junk" OR "sugary" OR "fizzy" OR "health" OR "sin" OR "demerit" OR "beverages" OR "drinks" OR "beverage" OR "drink") AND ("taxation" OR "taxes" OR "tax" OR "levy" OR "cess" OR "subsidy" OR "subsidies" OR "GST")]

**Articles assessed for eligibility**

1. Andersen, Henrik. “India–Solar Cells and Mexico–Taxes on Soft Drinks: Multilevel Rule of Law Challenges in the Interpretation of Art. XX (d) of GATT 1994 in WTO Case Law.” *Indian Journal of International Economic Law* 10, no. 1 (2018): 4.
2. Asia, South, Arun B Nair, Suresh K Mohammed, and Tackling Rising Obesity Rates. “The ‘Fat Tax’ in Kerala State, India: A Case Study,” 2019.
3. Badiani-Magnusson, Reena, and Katrina Jessoe. “Electricity Prices, Groundwater, and Agriculture: The Environmental and Agricultural Impacts of Electricity Subsidies in India.” In *Agricultural Productivity and Producer Behavior*, 157–83. University of Chicago Press, 2018.
4. Bagri, Neha Thirani. “India Stands Ground over Landmark WTO Deal; Pact to Ease Movement of Goods Is Held up by Dispute over Food Subsidy.” *International New York Times*, 2014, NA-NA.
5. Baig, Imran Ali, Muhammad Irfan, Md Abdus Salam, and Cem Işik. “Addressing the Effect of Meteorological Factors and Agricultural Subsidy on Agricultural Productivity in India: A Roadmap toward Environmental Sustainability.” *Environmental Science and Pollution Research* 30, no. 6 (2023): 15881–98.
6. Balamurugan, L. “Effect of GST on Agriculture-Input Market in India (A Survey Based Hypothetical Study).” *Think India Journal* 22, no. 10 (2019): 1148–58.
7. Bansal, CA Narendra Kumar, and Saurabh Sharma. “A Study on Impact of Goods and Services Tax on Agriculture Sector in India.” *Journal of Xidian University* 14, no. 5 (2020): 3797–3806.
8. Basu, Sanjay, Sukumar Vellakkal, Sutapa Agrawal, David Stuckler, Barry Popkin, and Shah Ebrahim. “Averting Obesity and Type 2 Diabetes in India through Sugar-Sweetened Beverage Taxation: An Economic-Epidemiologic Modeling Study.” *PLoS Medicine* 11, no. 1 (2014): e1001582.
9. Bathla, Seema, Pramod K Joshi, and Anjani Kumar. “Targeting Agricultural Investments and Input Subsidies in Low-Income Lagging Regions of India.” *The European Journal of Development Research* 31, no. 5 (2019): 1197–1226.
10. Bhardwaj, Upendra. “Agriculture Subsidies under WTO Order: A Case of Developing Nations with Reference to India and Food Security Act.” *Think India Journal* 22, no. 14 (2019): 11405–17.
11. Chakrabarti, Suman, Avinash Kishore, and Devesh Roy. “Effectiveness of Food Subsidies in Raising Healthy Food Consumption: Public Distribution of Pulses in India.” *American Journal of Agricultural Economics* 100, no. 5 (2018): 1427–49.
12. Danish, Muhammad Hassan, Muhammad Avais Tahir, Hafiz Syed M Azeem, and Mumtaz Anwar. “Impact of Agriculture Subsidies on Productivity of Major Crops in Pakistan and India,” 2017.
13. Deuss, Annelies, and Marcel Adenauer. “Food Subsidies in India: Comparing the Medium Term Impacts of Physical Grain Transfers and Different Types of Cash Transfers,” 2017.
14. Deveshwar, Aarti, and Ms Saloni. “Food Subsidy in India: A Conceptual Study.” *Journal of Scholastic Engineering Science and Management* 1, no. 6 (2022): 1–15.
15. Dixon, Peter, Maureen Rimmer, Rajesh Chadha, Devender Pratap, and Anjali Tandon. “Economy-Wide Analysis for India of the Effects of Removing Agricultural Subsidies,” 2015.
16. Dixon, Peter, Maureen T Rimmer, Rajesh Chadha, Devender Pratap, and Anjali Tandon. *A CGE Model for India with an Application on the Effects of Eliminating Agricultural Subsidies*. Centre of Policy Studies (CoPS), Victoria University, 2016.
17. Dizon, Felipe, and Jisang Yu. “Nudges versus Subsidies: Experimental Evidence on the Demand for a Nutritious Food in India.” *Journal of Economic Behavior & Organization* 191 (2021): 1091–1111.
18. Friedrich, MJ. “Tax on Sugary Beverages in India Could Reduce Obesity and Diabetes.” *JAMA* 311, no. 7 (2014): 666–666.
19. Gambhir, Ishita, Sonalde Desai, and Reeve Vanneman. “Role of Networks in the Elite Capture of Food Subsidy Program in India,” Vol. 29, 2017.
20. Gautam, Arun, Gitanjali Shrivastava, A Pankajam, Rohit Bansal, Ankur Gupta, and Malik Bader Alazzam. “A Study on Effects of GST on Farmers and Agricultural Sector: An Empirical Analysis in India,” Vol. 2587. AIP Publishing, 2023.
21. Gautam, Mr Arun, Saurabh Sharma, and CA Narendra Kumar Bansal. “To Study the Impact of Gabbar Singh Tax or Goods & Services Tax for Restaurant and Food Services Business in India,” n.d.
22. Gayatri, Yammanuru Phani, and Monish Jose. “Emission Taxes as a GHG Mitigation Mechanism in Agriculture: Effects on Rice Production of India.” *Agricultural Economics Research Review* 27, no. 2 (2014): 157–65.
23. Ghosh, Madhusudan. “Food Subsidy, Food Security and Hunger in India.” *Journal of Income & Wealth (The)* 37, no. 2 (2015): 64–74.
24. Ghoshal, Arunangshu, Edwin Saji, and Aju Mathew. “Income Tax Deduction as a Tool to Mitigate Catastrophic Health Expenditure for Cancer Care Falls Short of Its Purpose in India.” *Indian Journal of Medical Sciences*, 2024, 1–3.
25. Ginn, William, and Marc Pourroy. “The Contribution of Food Subsidy Policy to Monetary Policy in India.” *Economic Modelling* 113 (2022): 105904.
26. Hoda, Anwarul, and Prerna Terway. “Credit Policy for Agriculture in India: An Evaluation. Supporting Indian Farms the Smart Way. Rationalising Subsidies and Investments for Faster, Inclusive and Sustainable Growth.” Working Paper, 2015.
27. Husain, Zakir, Saswata Ghosh, and Mousumi Dutta. “Cash Transfers versus Food Subsidies during COVID-19: Dietary Practices of Rural Women in Bihar, India.” *Development in Practice* 33, no. 8 (2023): 986–1000.
28. Jain, Sandeep. “Taxation of Agriculture Income in India: An Overview.” *VISION: Journal of Indian Taxation* 2, no. 1 (2015): 95–104.
29. John, Rijo M, Fikru T Tullu, and Rachita Gupta. “Price Elasticity and Affordability of Aerated or Sugar-Sweetened Beverages in India: Implications for Taxation.” *BMC Public Health* 22, no. 1 (2022): 1372.
30. Joy, Justin. “Fertilizer Subsidy and Agricultural Production: A Study of India.” *Indian Journal of Economics and Development*, 2019, 1–7.
31. Jumrani, Jaya, and JV Meenakshi. “A Fat Subsidy and Its Impact on Edible Oil Consumption: Evidence from India.” Centre for Development Economics, Delhi School of Economics, 2020.
32. ———. “How Effective Is a Fat Subsidy? Evidence from Edible Oil Consumption in India.” *Journal of Agricultural Economics* 74, no. 2 (2023): 327–48.
33. Kaul, Tara. “Household Responses to Food Subsidies: Evidence from India.” *Economic Development and Cultural Change* 67, no. 1 (2018): 95–129.
34. Kishore, A. “Arbitrage and Corruption in Food Subsidy Programs: Evidence from India s Public Distribution System,” 2018.
35. Krishna, CJ, Dimple Shivani, and Jerlin Jose. “THE TAXATION ON AGRICULTURAL INCOME IN INDIA,” 2018.
36. Krishnakumar, V. “An Empirical Analysis on Food Subsidy in India.” *Humanities* 7, no. 1 (2019): 123–26.
37. Krishnamoorthy, Yuvaraj, Karthika Ganesh, and Manikandanesan Sakthivel. “Fat Taxation in India: A Critical Appraisal of Need, Public Health Impact, and Challenges in Nationwide Implementation.” *Health Promotion Perspectives* 10, no. 1 (2020): 8.
38. Kumar, Mannepalli Bharath. “Analysis of Growth Rates of Agricultural Subsidies and Capital Formation in Agriculture Sector of India,” 2022.
39. Kumar, Rajender, Ashima Sarin, and Somil Rastogi. “Bidi in Demerit Category (28%) in Goods Service Tax (GST) Was a Big Win in India.” *Tobacco Induced Diseases* 16, no. 1 (2018).
40. Kumar, Sanjeev. “Impact of Subsidies on Agriculture Sector in India.” *Agri Mirror: Future India1 (2)*, 2020, 40–44.
41. Kuriakose, Francis, and Deepa Kylasam Iyer. “Tax Landscape and Public Health: A Critical Examination of’Fat Tax’in Kerala, India.” *India (November 18, 2016)*, 2016.
42. LALVANI, MALA, and SWAHA SHOME. “INDIA’S BURGEONING FOOD SUBSIDIES: HOW MUCH CAN WE BLAME THE FOOD CORPORATION OF INDIA?” *International Economics/Economia Internazionale* 70, no. 3 (2017).
43. Law, Cherry, Kerry Ann Brown, Rosemary Green, Nikhil Srinivasapura Venkateshmurthy, Sailesh Mohan, Pauline FD Scheelbeek, Bhavani Shankar, Alan D Dangour, and Laura Cornelsen. “Changes in Take-Home Aerated Soft Drink Purchases in Urban India after the Implementation of Goods and Services Tax (GST): An Interrupted Time Series Analysis.” *SSM-Population Health* 14 (2021): 100794.
44. Mahajan, Mrs Meghana R, Sonali B Jadhav, and Mrs Sakshi Wani. “GST IN INDIA: FOOD AND AGRICULTURAL,” n.d.
45. Malaiarasan, Umanath, R Paramasivam, and K Thomas Felix. “Does Food Price Subsidy Affect Dietary Diversity? Evidence from South India.” *Margin: The Journal of Applied Economic Research* 15, no. 2 (2021): 268–90.
46. Malo, Mousumi. “The Overview of Government Subsidies to Agriculture Sector in India,” n.d.
47. Mandal, Soumya K, Arun Mitra, Yash Alok, Shubhanshu Gupta, and Anindo Majumdar. “Awareness and Perceptions Regarding Taxation and Health Warnings Related to Sugar-Sweetened Beverages and the Factors Associated with These among Visitors of a General out-Patient Clinic in Bhopal, India.” *Journal of Family Medicine and Primary Care* 9, no. 5 (2020): 2350–58.
48. Mowbray, Ash. “The Impact of High-Emission Food Subsidies on Global Warming in India,” n.d.
49. Mukhopadhyay, Indranil. “Tax Financed Health Insurance in India: Illusion of Financial Protection,” 2017.
50. Nair, Padmakumar Gokulkumar, and M Shamil. “Government Subsidies in India with Focus on Food Subsidy in View of the National Food Security Bill,” 2014.
51. Naschold, Felix, Leah EM Bevis, and Tanvi Rao. “The Individual and Intra-Household Effects of Food Price Subsidy Reform in Odisha (India),” 2018.
52. Oxford Analytica. “India Rejects Agriculture Tax despite Fiscal Impact.” *Emerald Expert Briefings*, no. oxan-db (2017).
53. Pandeya, Shinjini, Leah Bevisa, and Tanvi Raob. “Food Price Subsidies & Nutrition in India: Is Less Targeting More?,” n.d.
54. Panwar, Diksha, and Sidheswar Patra. “Impact of Goods and Services Tax on the Restaurants and Food Service Businesses in India.” *International Journal of Applied Business and Economic Research* 15, no. 23 (2017): 203–13.
55. Pearson, Daniel R. “India Tosses out the WTO’s Agricultural Subsidy Disciplines,” 2014.
56. Priyanka, P ASHA, and M Chandrasekaran. “Private and Social Cost of Use of Fertilizer and Drip System in Agriculture-a Study on’fertilizer and Drip Subsidy’in Sugarcane Cultivation in Tamil Nadu, India.” *International Research Journal of Agricultural Economics and Statistics* 6, no. 2 (2015): 232–41.
57. Ramaan Murthy, RV. “The Multiple Dimensions of Indian Agriculture and Its Political Economy: Political Economy of Agricultural Development in India-Policies, Achievements and Concernsby Akina Venkateswerlu, Aakar Books, and CESS, New Delhi, Pages 537, Price 1695,” 2022.
58. Ramappa, KB, and AV Manjunatha. “Agricultural Subsidies and Food Security: A Comparative Analysis of India and China.” *International Journal of Agriculture Sciences, ISSN*, 2016, 0975–3710.
59. Rana, Ritu. “How to Approach ‘Fat-Tax’on Food to Tackle Non-Communicable Diseases in India: A Review.” *Research Journal of Humanities and Social Sciences* 10, no. 2 (2019): 291–97.
60. Rege, Anushka, and Janice Ser Huay Lee. “State-Led Agricultural Subsidies Drive Monoculture Cultivar Cashew Expansion in Northern Western Ghats, India.” *PloS One* 17, no. 6 (2022): e0269092.
61. Reshamwala, Hussain S, Shivam Shrotriya, Bhaskar Bora, Salvador Lyngdoh, Rodolfo Dirzo, and Bilal Habib. “Anthropogenic Food Subsidies Change the Pattern of Red Fox Diet and Occurrence across Trans-Himalayas, India.” *Journal of Arid Environments* 150 (2018): 15–20.
62. Sahoo, Prangya P, Upasana Mohapatra, KK Sarangi, M Sangeetha, and Kirana L Kadam. “Impact of Gst on Agricultural Marketing in India.” *Indian Journal of Agricultural Marketing* 31, no. 3s (2017): 97–97.
63. Salunkhe, Harshal Anil. “Bureaucracy: A Study of Distribution Process of Agriculture Subsidy in India.” *International Journal Engineering Trends and Technology*, 2016, 5–8.
64. Salunkhe, Harshal Anil, and BB Deshmush. “Impact of Subsidy on Agriculture Sector in India-an Analytical Study.,” 2014.
65. ———. “The Sustainable Development of Agriculture Sector through Subsidies Is Possible in India.” *ZENITH International Journal of Business Economics & Management Research* 4, no. 2 (2014): 97–103.
66. SAMANTARA, DR RABINARAYAN. “Taxation of Agricultural Income in India.” *Journal Homepage: Http://Ijmr. Net. In* 9, no. 2 (2021).
67. Saraswat, SP, and ML Sharma. “AGRICULTURAL INPUT SUBSIDIES IN INDIA: QUANTUM OF SUBSIDIES TO SC/ST FRMERS-A STUDY IN HIMACHAL PRADESH,” n.d.
68. Sarva, Mahesh, Ajay Sidana, Rupesh Roshan Singh, and Gangu Naidu Mandala. “Revisiting State Subsidy Food Programme Model for India.” *International Journal of Recent Technology and Engineering* 8, no. 4 (2019): 2545–48.
69. Satapathy, Jyotirmayee, Narayan Chandra Nayak, and Jitendra Mahakud. “Investigating Preferences between Cash and In‐kind Transfers among the Food Subsidy Beneficiaries of India.” *Journal of Public Affairs* 22, no. 3 (2022): e2592.
70. Seema, Bathla, PK Joshi, and Kumar Anjani. “Revisiting Investments and Subsidies to Accelerate Agricultural Income and Alleviate Rural Poverty in India.” *IFPRI-Discussion Papers*, no. 1701 (2017).
71. Shah, Deepak. “Agricultural Subsidy in India: Flow of Public Expenditure, Issues and Concerns.” *Indian Journal of Agricultural Economics* 69, no. 3 (2014): 429.
72. ———. “Central Government Agricultural Subsidies in India: Public Sector Expenditure, Issues and Policy Implications,” 2021.
73. Sharma, Dinesh C. “Health Experts Welcome 40% Sin Tax on Tobacco in India.” *The Lancet Oncology* 17, no. 2 (2016): e49.
74. Shinde, Mr SB. “Agricultural Taxation In India.” *UGC Journal Details*, 2017.
75. Singh, Arunima, and Parvati Pyarelal. “Is Fat Tax the Panacea for Fast Food Consumption and Related Health Issues in Urban India?,” 2019.
76. Singh, Naveen P, Jaiprakash Bisen, P Venkatesh, and KS Aditya. “GST in India: Reflections from Food and Agriculture.” *Agricultural Economics Research Review* 31, no. 2 (2018): 175–85.
77. Sree, Shilpa, and Basavaraj S Benni. “IMPACT OF AGRICULTURAL SUBSIDIES ON PRODUCTION AND PRODUCTIVITY IN INDIA,” n.d.
78. Tandon, Anjali, and Roopali Aggarwal. “Evaluating the Role of Subsidies in Sustainable Agriculture: A Case Study of India.” In *Indian Agriculture Under the Shadows of WTO and FTAs: Issues and Concerns*, 161–76. Springer, 2021.
79. Thomas, Ashok, Kausik Gangopadhyay, Kulbhushan Balooni, Satheeshan Balasubramanian, and Maya Padmanabhan. “Is Government Health Subsidy Targeted Enough in India? Estimation of Private and Public Treatment Costs for Cancer Patients.” *Working Papers*, Working papers, March 2022. [https://ideas.repec.org//p/iik/wpaper/523.html](https://ideas.repec.org/p/iik/wpaper/523.html).
80. ———. “Is Government Health Subsidy Targeted Enough in India? Evidence from a Tertiary Hospital.” *International Journal of Public Administration*, 2024, 1–21.
81. Thomas, Sweety. “The Burden of Food Subsidy and Mounting Stocks of Food Grains in India: Empirical Analysis.” *South Asian Journal of Marketing & Management Research* 7, no. 10 (2017): 49–64.
82. Trumboo, Naveed. “Agricultural Income Tax in India: Introducing Threshold-Based Tax Exemption.” *Indian Journal of Public Administration* 68, no. 1 (2022): 21–33.
83. Varghese, Beena, Rajashree Panicker, Dripto Mukhopadhyay, Kathryn Backholer, Vani Sethi, Arjan de Wagt, Zivai Murira, Neena Bhatia, and Monika Arora. “Estimating the Potential Impact of a Health Tax on the Demand for Unhealthy Food and Beverages and on Tax Revenue in India.” *Health Policy and Planning* 39, no. 3 (2024): 299–306.
84. Venkatraja, B. “Is Rising Food Subsidy Attributed to Poor Food Management? An Empirical Evidence from India.” *International Affairs and Global Strategy* 51 (2016): 15–24.
85. Vishal, J. “Agricultural Subsidies in India–An Overview.” *Thematics Journal of Geography* 8, no. 12 (2019): 482–86.
86. Yadav, Mohit. “Taxation on Agricultural Income in India.” *Issue 2 Indian JL & Legal Rsch.* 5 (2023): 1.
87. Zafar, Shadman, Mohammad Aarif, and Md Tarique. “Input Subsidies, Public Investments and Agricultural Productivity in India.” *Future Business Journal* 9, no. 1 (2023): 54.
88. Zafar, Shadman, and Md Tarique. “Efficacy of Public Spending for Agricultural Development in India: A Disaggregate Analysis Contextualizing Subsidies vs Investment Debate.” *International Journal of Social Economics* 50, no. 7 (2023): 925–40.
89. Zaki, M, H Adolf, A Chandrawulan, and S Dewi. “Public Stockholding Regulations and Domestic Agricultural Subsidies in China, India, Brazil and Indonesia to Achieve Food Security in Sustainable Development Goals,” 2019.

Appendix B: Evidence Map
***Table S1: Extraction map of included studies and documents by health policy triangle dimension***

| **Author (Year)** | **Fiscal instrument** | **Commodity** | **Content** | **Context** | **Process** | **Actors** |
| --- | --- | --- | --- | --- | --- | --- |
| Jumrani & Meenakshi (2020) | Subsidy | Edible oils | x |  |  |  |
| Basu, Vellakal, Agrawal, Stuckler, Popkin & Ebrahim (2014) | Taxes | SSB |  | x |  |  |
| Mandal, Mitra, Alok, Gupta & Majumdar (2020) | Taxes | SSB |  | x |  | x |
| Husain, Ghosh & Dutta (2023) | Subsidy | PDS |  |  |  |  |
| Law, Brown, Green, Venkateshmurthy, Mohan, Scheelbeek, Shankar, Dangour, Cornelsen (2021) | Taxes | SSB | x |  |  |  |
| Malaiarasan, Paramasivam & Felix (2021) | Subsidy | Rice | x |  |  |  |
| Chakrabarti, Kishore & Roy (2018) | Subsidy | Pulses | x |  |  |  |
| Varghese, Panicker, Mukhopadhyay, Backoler, Sethi, de Wagt, Murira, Bhatia & Arora (2024) | Taxes | Sugar, SSBs, HFSS foods | x |  |  |  |
| Krishnamoorthy, Ganesh & Sakthivel (2020) | Taxes | Fat |  |  | x |  |
| Pandeya, Bevisa & Raob (2022) | Subsidy | Public Distribution System items | x |  |  |  |
| Singh, Bisen, Venkatesh & Aditya (2018) | Taxes | Agricultural produce |  |  | x |  |
| Kaul (2018) | Subsidy | Public Distribution System items |  |  | x |  |
| Rana (2019) | Both | Broad categories of healthy and unhealthy foods | x |  |  |  |
| Panwar & Patra (2017) | Taxes | Prepared meals, snacks and beverages by food service businesses | x |  |  |  |
| Singh & Pyarelal (2019) | Taxes | Fat | x |  |  |  |
| Venkatraja (2016) | Subsidy |  |  |  | x |  |
| Dizon & Yu (2021) | Subsidy | Fortified food |  | x |  |  |
| John, Tullu, Fikru & Gupta (2022) | Taxes | SSBs; Aerated beverages |  | x |  |  |
| Kuriakose & Iyer (2016) | Taxes | Fat, SSBs | x |  |  |  |
| Nair & Mohammed (2019) | Taxes | Fat | x |  |  |  |
| Gautam, Sharma & Bansal (2019) | Taxes | Prepared meals, snacks and beverages by food service businesses |  |  |  | x |

*Note:* "x" indicates that the study or document addresses the corresponding health policy triangle dimension. Abbreviations: HFSS, high in fat, sugar, and salt; PDS, public distribution system; SSB, sugar-sweetened beverage.

Appendix C: Other Stakeholders Identified for Collaboration
***Table S2: Additional stakeholders identified through the scoping review and key informant interviews***

Agricultural and Processed Food Products Export Development Authority

Allied Health Professions Council

Federation of Hotel & Restaurant Associations of India

Food Corporation of India

Indian National Committee of International Commission for Uniform Methods of Sugar Analysis (ICUMSA)

Indian Society of Lifestyle Medicine

Legal Metrology

Medical Council of India

Ministry of Ayurveda, Yoga and Naturopathy, Unani, Siddha and Homeopathy

Ministry of Commerce and Industry

Ministry of Consumer Affairs, Food and Public Distribution

Ministry of Education (Department of School Education)

Ministry of Food and Civil Supplies

Ministry of Information and Broadcasting

National Commission for Protection of Child Rights

National Institute of Food Technology, Entrepreneurship and Management

NITI Aayog

Startup India

Telecom Regulatory Authority of India

*Note:* Abbreviations: ICUMSA, International Commission for Uniform Methods of Sugar Analysis; NITI, National Institution for Transforming India.
